# The Treatment of Gout

**Published:** 1903-09-12

**Authors:** 


### THE TREATMENT OF GOUT.

In the treatment of gouty arthritis Dr. London,[1] of Carlsbad, points out that prophylaxis is of paramount importance. Hence the diet must be strictly regulated and all excess in food and drink avoided, the bowels regulated, and a proper amount of bodily work and exercise undertaken so as to increase the circulation and favour the excretion of waste products. To avoid overloading the organism with uric acid not only must its formation be prevented, but its elimination must be encouraged by accelerating its further oxidation, by increasing its solubility in the blood and tissue-juices and by aiding excretion. The important part played by the digestive apparatus in arthritic processes and uric-acid metabolism justifies the assumption that the course of gout may be influenced through the agency of the alimentary canal, and it is therefore common to prescribe a saline aperient in commencing a "cure," more especially if habitual constipation is present. The excretion of uric acid by the kidneys is encouraged by the copious administration of fluids, or by means which influence the circulation of the blood. An endeavour may be made to raise the uric acid elimination by oxygen inhalation, by the ingestion of thyroid extract, or by the use of spermin. Since uric acid is deposited in the gouty areas in a form which is not easily soluble, attempts may be made to remove these deposits by inducing more favourable conditions for their solution. Thus attempts have been made to increase the alkalinity of the blood and tissue-juices by the administration of alkali salts of vegetable acids, but it has been found under such circumstances that the excess of alkalies is rapidly thrown out in the urine. Again, lithium salts, which in laboratory experiments form easily soluble salts with uric acid, have failed to be of much value clinically, and no greater success has attended the administration of organic nitrogenous bases such as piperazin and lysidin. They appear to be incapable of dissolving uric acid in the body. It is hoped that some success may attend the endeavour to produce combinations between uric acid and other compounds, such as formaldehyde. For this purpose urotropine is given, which liberates formaldehyde when taken into the body. Experience has shown that suitable dieting has achieved greater results than any other method of treatment. During an acute attack, only such food should be allowed as is incapable of producing free uric acid, and this requirement is fulfilled by the carbohydrates. It is well also to remember that vegetable albumen contributes little, if at all, to the formation of uric acid. Fruit, with the exception of strawberries and raspberries, need not be prohibited. Eggs may be allowed, since the white is free from nuclein, while the yolk only contains a paranuclein, which is incapable of giving rise to uric acid. Milk is useful and harmless. Meat and meat soups should be avoided entirely at first, and in gouty patients at all times the amount of food taken should be regulated. Alcohol in any case ought to be limited to the smallest quantity only, or withheld altogether. Next in importance to diet is a rational mode of life and exercise in the open air. Among drugs, colchicum and its compounds are of most value during the acute stage. Colchicum influences the renal secretion as well as intestinal activity, diminishes pain, and, lastly, is said to modify the abnormal processes which underlie the gouty condition. Dr. London usually employs the tincture or the vinum, the latter in 10 to 20 minim doses several times a day up to 100 minims. If the acute attack persists, he finds a combination of potassium iodide and colchicum of great use. Salicylates act in gout, as in rheumatism, by relieving pain, but at the same time they also cause a diminution of the swelling. Of the preparations of salicylic acid, aspirin, in doses of one gramme hourly during the day and four to five grammes at night has been effective. In troublesome cases, where no effect is produced by aspirin in the course of a few days, antipyrin, salipyrin, or phenacetin may be substituted. As soon as the pain is even slightly relieved, Dr. London allows the patient to leave his bed, and encourages walking exercise as early as possible. This treatment he states will shorten an attack that would last for weeks if the patient were kept in bed. In all stages of gout alkaline medication is indicated, though alkaline waters ought to be sparingly taken so as to avoid overloading the body with alkalies, since an excess of alkali, especially bicarbonate and carbonate of sodium, may directly induce a deposition of biurates in the tissues.

[1] Practitioner, Sept., 1903.

